# Novel ultrasound classification of tubal ectopic pregnancy: exploring underlying connections among sonographic and serum markers

**DOI:** 10.1186/s13244-025-02079-2

**Published:** 2025-09-17

**Authors:** Na Su, Lin-Ru Fu, Zhe Du, Hua-Zhen Liu, Zhao Tian, Tao Xu, Zi-Jing Fu, Zhen-Hong Qi, Zhi-Jing Sun

**Affiliations:** 1 Department of Ultrasound, Peking Union Medical College Hospital, Chinese Academy of Medical Sciences & Peking Union Medical College, Beijing, China; 2 Department of Obstetrics and Gynecology, Peking Union Medical College Hospital, Chinese Academy of Medical Sciences & Peking Union Medical College, National Clinical Research Center for Obstetric & Gynecologic Diseases, Beijing, China; 3 Department of Epidemiology & Biostatistics, Institute of Basic Medical Sciences, Chinese Academy of Medical Sciences & School of Basic Medicine, Peking Union Medical College, Beijing, China

**Keywords:** Ectopic pregnancy, Human chorionic gonadotropin, Ultrasonography, Hemoglobin

## Abstract

**Objectives:**

This study aims to propose a novel ultrasound classification for tubal ectopic pregnancy (TEP) and explore relationships between sonographic features and serum markers.

**Materials and methods:**

A retrospective single-center cohort study was conducted involving TEP patients, who were classified into two groups (simple gestational sac (GS)-like and complicated masses) based on ultrasound characteristics indicating a clear trophoblastic ring, hematosalpinx, or clear salpinx structure. Statistical comparisons and Spearman’s rank correlation analyses were performed.

**Results:**

In this study, 320 women with TEP were classified into a simple GS-like mass group (*n* = 128) and a complicated mass group (*n* = 192). Compared with complicated masses, simple GS-like masses were smaller, had higher β-human chorionic gonadotropin (β-hCG) levels, and higher color Doppler flow imaging grades (all *p* < 0.05). β-hCG was strongly correlated with mass size in simple GS-like mass cases (*r* = 0.720, *p* < 0.01), but weakly correlated in complicated mass cases (*r* = 0.211, *p* < 0.01). Hemoglobin levels were inversely correlated with pelvic free fluid depth in complicated masses (*r* = −0.344, *p* < 0.01).

**Conclusions:**

The proposed ultrasound classification differentiates TEPs into subtypes with unique clinical and pathophysiological profiles. The variation in the correlation among sonographic indices and serum markers across TEP subtypes underscores the necessity for tailored clinical strategies. In simple GS-like masses, β-hCG and mass size are positively correlated, thus, clinical decisions could be made based on these two factors as recommended by guidelines. However, in complicated masses, β-hCG and mass size were poorly correlated, thus, the mass size should be deemphasized. Decisions should be made through comprehensive assessments under such circumstances.

**Critical relevance statement:**

The novel ultrasound-based classification proposed in this study differentiates tubal ectopic pregnancies into simple gestational-sac-like and complicated subtypes presenting with different correlations between sonographic indices and serum markers, which require tailored strategies in clinical settings.

**Key Points:**

- Addresses the lack of unified ultrasound classification for TEP.
- Different TEP types reveal distinct sonographic and serum profiles, with various relationships between them.
- This novel classification aids in tailored treatment and clinical decision-making.

**Graphical Abstract:**

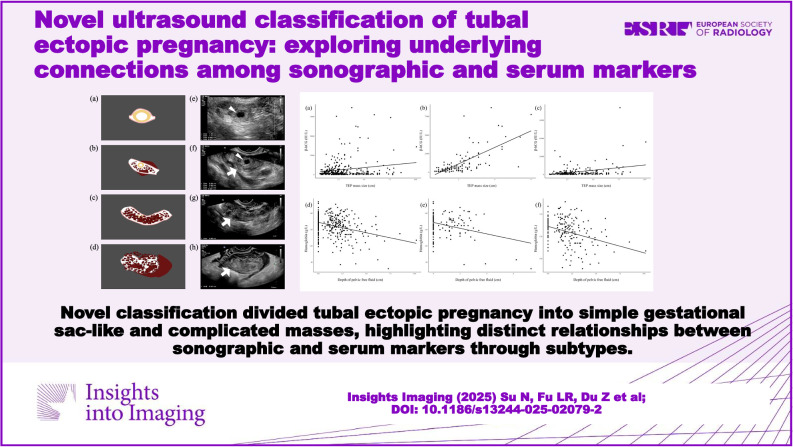

## Introduction

Ectopic pregnancy (EP) accounts for approximately 2% of all pregnancies, and 18% of patients who visit the emergency department with early-pregnancy-related abdominal pain or vaginal bleeding are diagnosed with EP [1]. Among these, tubal ectopic pregnancy (TEP) is the most common type [2]. Management of TEP involves medical, surgical, or expectant approaches, with decisions depending on comprehensive clinical information.

Current guidelines incorporate adnexal mass size and β-hCG levels into treatment algorithms, but recommendations differ [1, 3, 4]. The 2023 National Institute for Health and Care Excellence guideline advised surgery as first-line management for any TEP with an adnexal mass ≥ 3.5 cm, irrespective of β-hCG level [3]. In contrast, the American College of Obstetricians and Gynecologists considers a mass > 4 cm a relative, rather than absolute, contraindication to methotrexate therapy, without specifying absolute β-hCG cutoff values [1]. This variability underscores the clinical challenge posed by discordant mass sizes and β-hCG levels, which often arise because TEPs present diverse sonographic appearances [5]. The absence of a unified ultrasound classification further hinders the integration of sonographic findings with biochemical markers [6–8]. Categorizing these ultrasound patterns offers a potential solution to address this discrepancy and guide more precise management of this condition.

Therefore, in this study, a novel morphology-based ultrasound classification is proposed to address these gaps. Using this new classification, we aim to further analyze the relationships among serum markers and sonographic indices, which may provide new insights into the individualized management of TEP.

## Methods

### Patient recruitment and ethics

This retrospective observational study was conducted in accordance with the Declaration of Helsinki (revised in 2013), and it was approved by the Institutional Review Board of the Peking Union Medical College Hospital (PUMCH) (NO.I-24PJ0349). Informed consent was waived due to the retrospective observational setting. No additional information or samples were collected beyond medical record data and anonymized ultrasound images, and all sensitive information was removed during data collection.

**Fig. 1:**
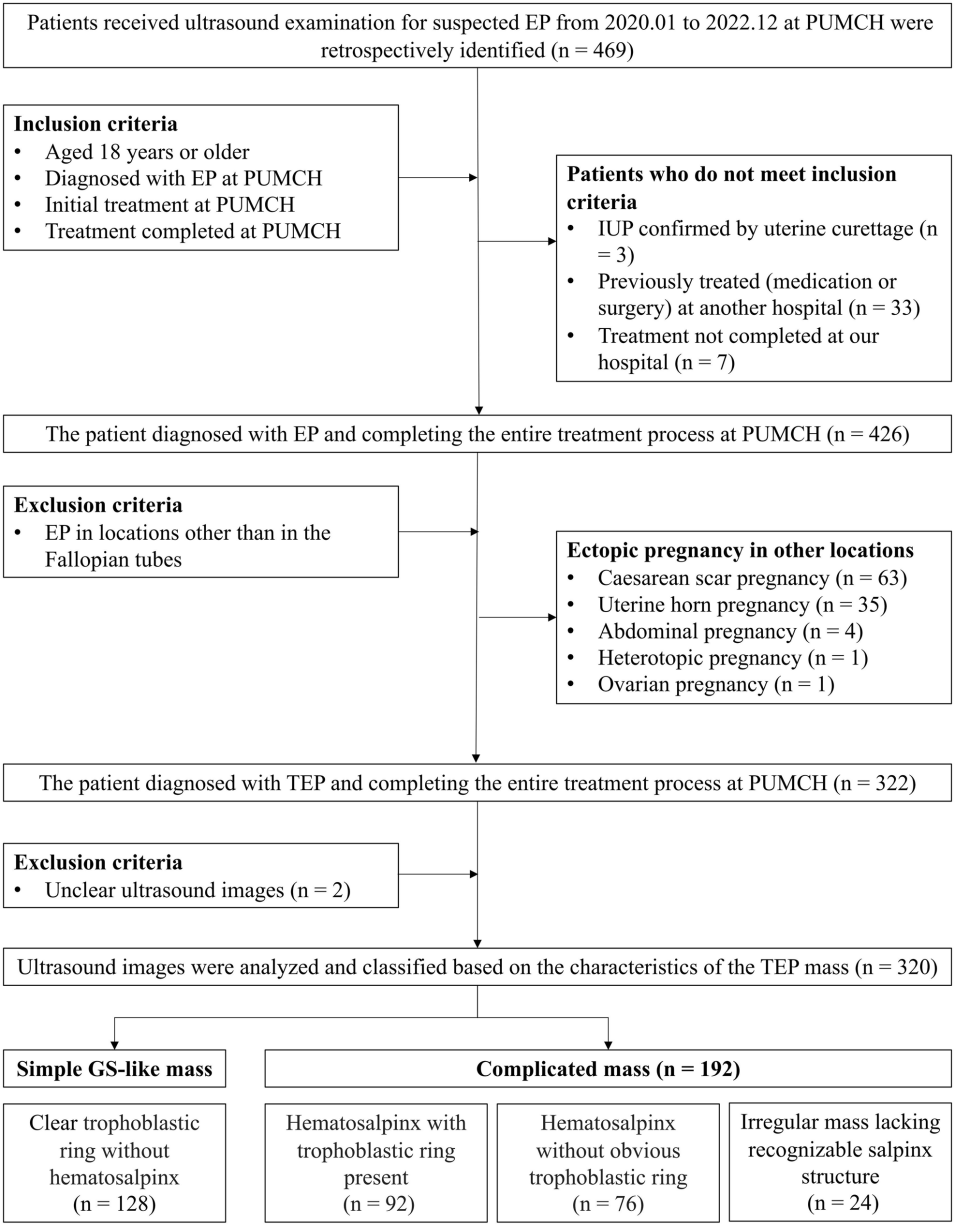
Flow diagram of patient selection. EP, ectopic pregnancy; PUMCH, Peking Union Medical College Hospital; IUP, intrauterine pregnancy (either live or not); TEP, tubal ectopic pregnancy; GS, gestational sac

Female patients who underwent ultrasound examinations for suspected EP between January 2020 and December 2022 at PUMCH were retrospectively and consecutively screened (Fig. 1). The inclusion criteria for patients were as follows: (1) 18 years or older; (2) diagnosed with EP at PUMCH on the basis of a comprehensive analysis of clinical information; (3) treated initially at PUMCH with no prior intervention for EP; and (4) treated entirely at PUMCH. Patients were excluded if they (1) were finally diagnosed with EP in locations other than TEP or (2) lacked clear ultrasound images for visualization of the adnexal mass. Patients were diagnosed with TEP by a comprehensive analysis of symptoms, ultrasound features, serum β-hCG measurements, negative uterine aspiration when performed, and laparoscopic and histopathological confirmation where applicable. In medically or expectantly managed patients, diagnostic confirmation also required a reduction, and eventual disappearance, of the adnexal mass on serial ultrasound when β-hCG decreases to negative levels, which reflects the expected course of TEP.

### Clinical definitions

The number of previous EPs was denoted as the previous EP. β-hCG and hemoglobin measurements were obtained within 24 h of the ultrasound examination. If multiple measurements were performed within that window, the result nearest the ultrasound time was selected for analysis. No therapeutic interventions were administered before measurements were taken. The management strategy was chosen for TEP patients on the basis of clinical guidelines [1, 3, 4], as mentioned in our previous study [9]. All clinical decisions were made in collaboration with patients. Some patients might not accept treatment recommended by evidence-based medicine, but all patients received full-course management at our center and received follow-up until β-hCG tests became negative.

### Ultrasonographic definitions

Most patients underwent transvaginal ultrasound, with a few patients undergoing transabdominal ultrasound due to limitations at the time of examination (such as pain, heavy vaginal bleeding, etc.). All ultrasounds were performed using a commercially available EPIQ7 system (Philips Medical Systems) equipped with high-resolution probes at frequencies of 1–5 MHz for abdominal imaging and 3–10 MHz for transvaginal imaging, which is a standard clinical equipment at PUMCH, by multiple well-trained radiologists with professional ultrasound certifications and at least 3 years of clinical experience in ultrasonography, in the Department of Ultrasound at PUMCH and clear ultrasound images were retained. All images were reviewed and analyzed by two radiologists (N.S. and Z.-H.Q.) who have over 15 years of experience in gynecological ultrasonography at PUMCH, and if there was any disparity, further discussions were conducted to reach a consensus.

**Fig. 2:**
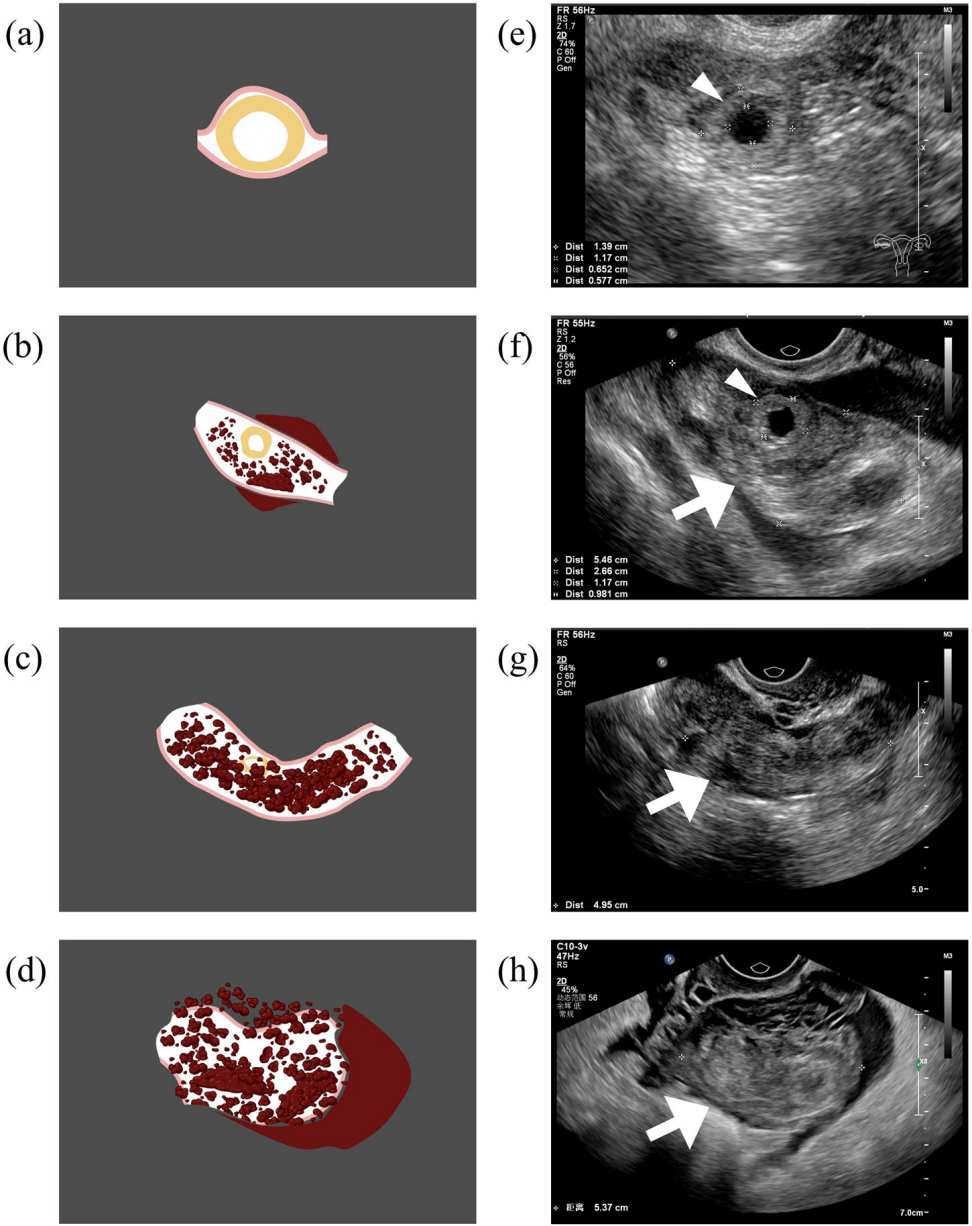
Classification of TEP masses on the basis of ultrasonographic features. TEP masses are categorized into simple GS-like masses and complicated masses, as illustrated in the ultrasound images: (**e**) simple GS-like masses include a clear trophoblastic ring (arrowhead) without hematosalpinx. Complicated masses include three manifestations: (**f**) a hematosalpinx (arrow) with a trophoblastic ring (arrowhead), which appears as a sausage-shaped hypoechoic structure (distended by blood) containing a trophoblastic ring; (**g**) a hematosalpinx (arrow) without an obvious trophoblastic ring, which manifests as a sausage-shaped hypoechoic structure (distended by blood) without a recognizable trophoblastic ring; and (**h**) an irregular mixed-echoic mass (arrow), which is seen as an adnexal mass lacking recognizable tubal structure with or without a visible GS structure inside. Example images presented herein were all obtained through grayscale transvaginal ultrasound scanning. **a**–**d** correspond to the respective schematic representations of the ultrasound patterns in **e**–**h**. TEP, tubal ectopic pregnancy; GS, gestational sac

On the basis of the ultrasonographic characteristics of the TEP masses [5, 8], they were categorized into 2 types as follows: simple gestational sac (GS)-like masses, which indicated a clear trophoblastic ring (variable echogenicity, often with its wall appearing more echogenic than the endometrium and enclosing a central anechoic area [1, 10–12]) without hematosalpinx (Fig. 2e); and complicated masses, including three manifestations: (1) a hematosalpinx with a trophoblastic ring present (Fig. 2f), which appeared as a sausage-shaped hypoechoic structure (distended by blood) containing a trophoblastic ring; (2) a hematosalpinx without an obvious trophoblastic ring (Fig. 2g), which manifested as a sausage-shaped hypoechoic structure (distended by blood) without a recognizable trophoblastic ring; or (3) an irregular mixed-echoic mass (Fig. 2h), which was observed as an adnexal mass lacking a recognizable tubal structure with or without a visible GS structure inside. In Fig. 2a–d correspond to the respective schematic representations of these ultrasound patterns in (e–h). Real-time ultrasound videos of each TEP subtype are provided in Videos S1–S4, with corresponding static images in Figs. S1–S4. Each TEP mass was measured along three perpendicular axes, namely, the longitudinal diameter on the maximal longitudinal plane, and the transverse and anteroposterior diameters on the maximal transverse plane, following a protocol proposed by the ESHRE working group on EP [5, 8], and the maximum diameter was recorded as the TEP mass size. For simple GS-like masses, the outer-to-outer margins of the trophoblastic ring were measured. For complicated masses, when there was a hematosalpinx with or without a trophoblastic ring, the inner-to-inner margins of the salpinx were measured; when the salpinx structure was absent, the full size of the adnexal mass was measured.

**Fig. 3:**
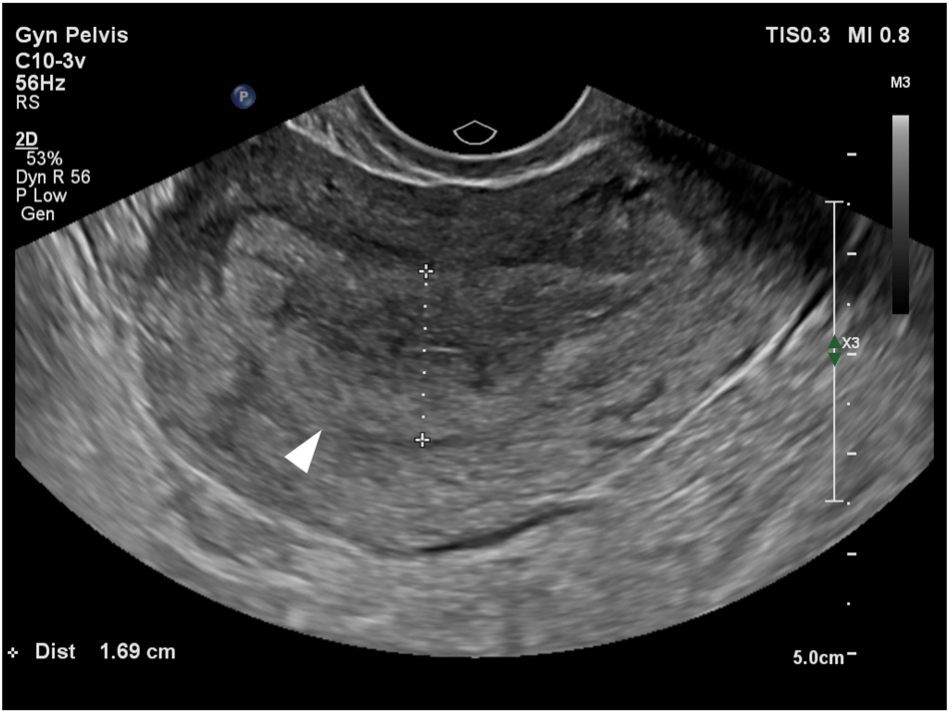
An example of an endometrial thickness measurement via transvaginal ultrasound. The uterine sagittal view shows the endometrial echo (arrowheads) extending from the fundus to the cervix. A dashed caliper line is placed perpendicular to the endometrial midline, with endpoints aligned at the outer borders of the endometrium, which yields a thickness of 1.69 cm

Pelvic free fluid was measured by the anteroposterior depth from ultrasound images. Endometrial thickness was measured by identifying the sagittal view of the uterus, locating the midline view with the endometrium visible from the fundus to the cervix, positioning a line perpendicular to the midline, and measuring the thickness by aligning the endpoints with the outer edges of the endometrium (Fig. 3).

**Fig. 4:**
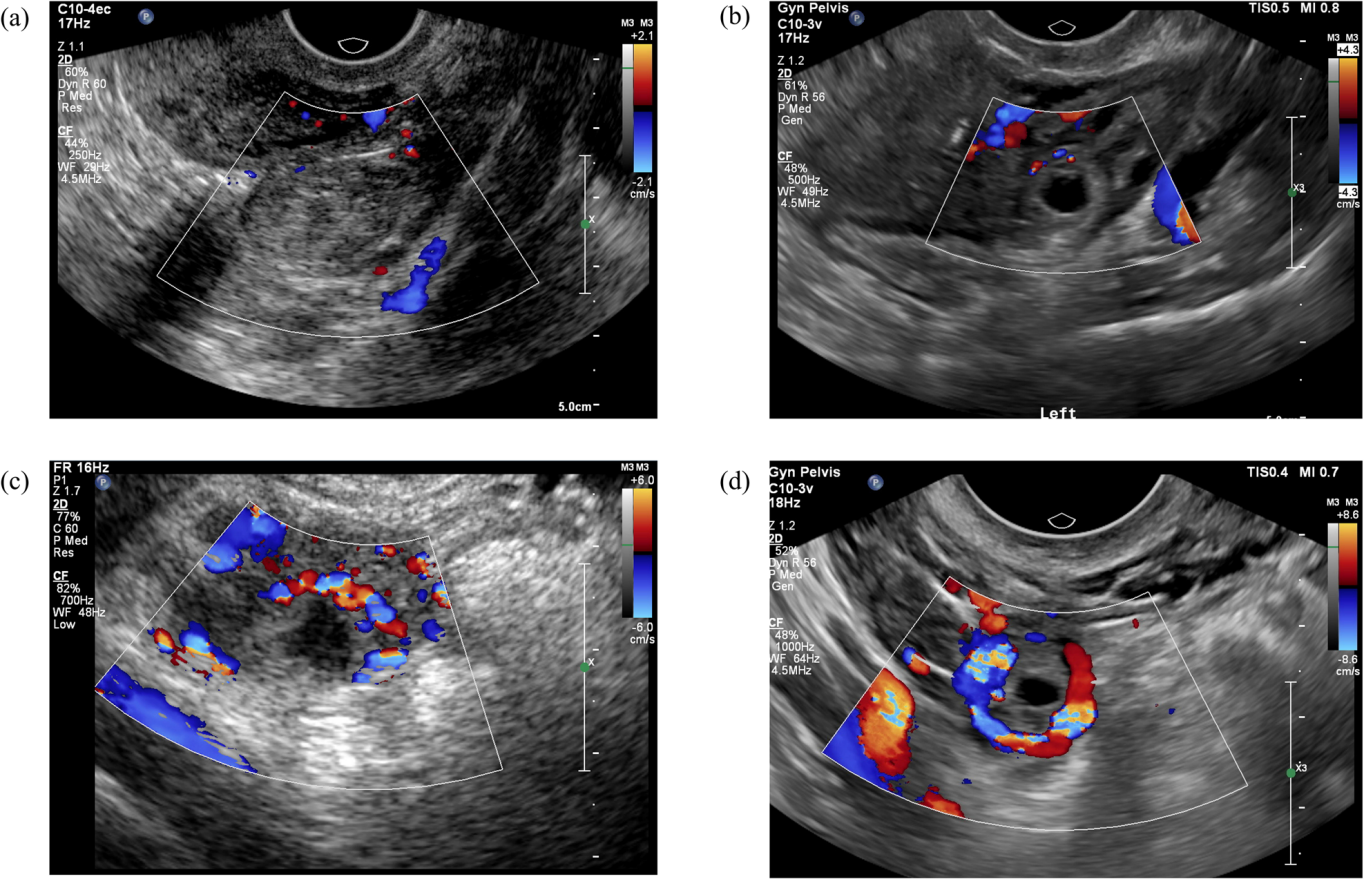
Color Doppler flow image grading in tubal ectopic pregnancies. (**a**) grade 0: no vascularity; (**b**) grade 1: vascularity occupying less than one third of the trophoblastic ring or suspected pregnancy mass; (**c**) grade 2: vascularity occupying more than one third but less than two thirds of the trophoblastic ring or suspected pregnancy mass; (**d**) grade 3: vascularity occupying more than two thirds of the trophoblastic ring or suspected pregnancy mass. The example images provided here were all obtained via transvaginal ultrasound

Peri-trophoblastic blood flow varied among patients and was assessed using color Doppler flow imaging (CDFI) [10]. CDFI was graded on a scale from 0 to 3 (Fig. 4), which is clinically recognized and routinely practiced at PUMCH, and was previously described by Atri et al [13] as follows: grade 0 refers to no vascularity; grade 1 refers to vascularity occupying less than 1/3 of the trophoblastic ring or suspected pregnancy mass; grade 2 refers to vascularity occupying 1/3 to 2/3 of the trophoblastic ring or suspected pregnancy mass; and grade 3 refers to vascularity occupying more than 2/3 of the trophoblastic ring or suspected pregnancy mass. Misinterpretation of tubal blood flow as peri-trophoblastic blood flow was avoided, especially in complicated masses, where blood flow of the wall of the salpinx located at the outermost margin of the mass and separated from gestational tissue by a clot, must not be mistaken for peri-trophoblastic blood flow, and true intralesional flow within the gestational tissue should be confirmed carefully (for example, as shown in Fig. S5).

### Statistical analysis

Simple GS-like and complicated mass groups were compared in this study. Continuous variables were tested for a normal distribution using Kolmogorov–Smirnov test. Variables following a normal distribution are presented as the mean and standard deviation, whereas those not normally distributed are presented as the median and interquartile range (25–75th percentiles). Categorical variables are presented as counts and percentages. The mean or median differences were evaluated using *t*-test or a Wilcoxon rank-sum test, respectively. When comparing categorical variables, the chi-squared test was applied when the expected frequency in all cells was greater than or equal to 5, otherwise, Fisher’s exact test was used. Statistical significance was set at a two-sided *p* < 0.05. Spearman’s rank correlation with a two-sided *p*-value of 0.05 was used to derive correlation coefficients between serum markers and sonographic indices. Statistical analyses were performed by L.-R.F., H.-Z.L., and T.X. via R software version 4.3.3 (R Foundation for Statistical Computing).

## Results

### Population characteristics

As reported in the flowchart (Fig. 1), 469 patients were initially considered for inclusion, with 320 enrolled for analysis. According to their ultrasound characteristics, 128 (40.0%) and 192 (60.0%) masses were classified as simple GS-like and complicated masses, respectively. The patient characteristics are summarized in Table 1, with 6 (1.9%) undergoing transabdominal ultrasound and 314 (98.1%) undergoing transvaginal ultrasound. There were no significant differences in age, gravidity, parity, previous EP counts, body mass index, gestational age, conception method, or luteal support history (*p* > 0.05) between the simple GS-like and the complicated mass group. In terms of symptoms, there was no difference in abdominal pain between the two groups (*p* = 0.07), whereas vaginal bleeding was more common in complicated patients (89.1% vs 78.1%, *p* = 0.01).

**Table 1:** Characteristics of patients with different types of TEP masses

Variables	Total cohort (*n* = 320)	Simple GS-like mass (*n* = 128)	Complicated mass (*n* = 192)	*p*
Age (years)	32.00 (29.00–36.00)	32.00 (30.00–36.00)	32.00 (29.00–36.00)	0.22
Gravidity	2.00 (1.00–3.00)	2.00 (1.00–3.00)	2.00 (1.00–3.00)	0.80
Parity	0.00 (0.00–1.00)	0.00 (0.00–1.00)	0.00 (0.00–1.00)	0.94
Previous EP	0.00 (0.00–0.00)	0.00 (0.00–0.00)	0.00 (0.00–0.00)	0.32
BMI (kg/m^2^)	20.75 (19.33–23.05)	20.90 (19.56–23.71)	20.70 (19.05–22.66)	0.09
Gestational age (days)	45.50 (40.00–53.00)	45.00 (40.00–51.25)	47.00 (41.00–53.00)	0.26
Conception method				0.57
ART	13 (4.1)	4 (3.1)	9 (4.7)	
Natural	307 (95.9)	124 (96.9)	183 (95.3)	
Luteal support	33 (10.3)	14 (10.9)	19 (9.9)	0.85
Vaginal bleeding	271 (84.7)	100 (78.1)	171 (89.1)	**0.01**
Abdominal pain	220 (68.8)	80 (62.5)	140 (72.9)	0.07
β-hCG (IU/L)	2244.50 (905.73–6008.62)	5455.95 (2536.35–12,174.42)	1280.10 (602.46–2677.57)	**< 0.01**
Hemoglobin (g/L)	126.00 (118.00–133.00)	127.00 (119.00–133.00)	126.00 (117.75–132.00)	0.18
Ultrasound type				1.00
Transabdominal	6 (1.9)	2 (1.6)	4 (2.1)	
Transvaginal	314 (98.1)	126 (98.4)	188 (97.9)	
Ultrasound characteristics				
Pregnancy location				0.09
Left	171 (53.4)	76 (59.4)	95 (49.5)	
Right	149 (46.6)	52 (40.6)	97 (50.5)	
TEP mass size (cm)	2.40 (1.67–3.90)	1.80 (1.30–2.50)	3.00 (2.20–4.80)	**< 0.01**
CDFI grading				**< 0.01**
0	38 (11.9)	0 (0.0)	38 (19.8)	
1	131 (40.9)	27 (21.1)	104 (54.2)	
2	93 (29.1)	54 (42.2)	39 (20.3)	
3	58 (18.1)	47 (36.7)	11 (5.7)	
Positive fetal heartbeat	28 (8.8)	25 (19.5)	3 (1.6)	**< 0.01**
Endometrial thickness (cm)	1.00 (0.60–1.40)	1.20 (0.80–1.60)	0.80 (0.50–1.30)	**< 0.01**
Depth of pelvic free fluid (cm)	1.40 (0.00–2.40)	0.00 (0.00–1.60)	1.80 (0.85–2.80)	**< 0.01**

Data are presented as mean ± SD, *n* (%), or median (interquartile range). *p*-values < 0.05 are shown in bold

*TEP* tubal ectopic pregnancy, *GS* gestational sac, *EP* ectopic pregnancy, *BMI* body mass index, *ART* assisted reproductive technology, *β-hCG* β-human chorionic gonadotropin, *CDFI* color Doppler flow imaging

For serum markers, although there was no significant difference in hemoglobin levels between the two groups (*p* = 0.18), the complicated mass group presented lower β-hCG levels than the simple GS-like group did (1281.10 IU/L vs 5455.95 IU/L, *p* < 0.01). When the ultrasound characteristics were compared, the TEP mass size was smaller in the simple GS-like mass group (1.80 cm vs 3.00 cm, *p* < 0.01), with higher CDFI grading and a more frequent presence of a fetal heartbeat (*p* < 0.01). Additionally, the endometrium was significantly thicker in the simple GS-like mass group than in the complicated mass group (1.20 cm vs 0.80 cm, *p* < 0.01), whereas the pelvic free fluid was significantly deeper in the complicated mass group (1.80 cm vs 0.00 cm, *p* < 0.01).

Management differed significantly between the two groups (*p* < 0.01). Overall, 24 patients (7.5%) were managed expectantly, 91 (28.4%) were treated with methotrexate alone, 162 (50.6%) were managed by laparoscopic surgery alone, and 43 (13.4%) received combined laparoscopic surgery and methotrexate treatment. The complicated mass group had higher rates of expectant management (10.4% vs 3.1%) and methotrexate monotherapy (36.5% vs 16.4%), whereas the simple GS-like group more frequently underwent surgery with (18.0% vs 10.4%) or without (62.5% vs 42.7%) methotrexate. There was no significant difference in tubal rupture rates between the two groups (7.0% vs 10.4%, *p* = 0.33). Notably, in the simple GS-like mass group without detectable fetal heartbeats (*n* = 103), 100 patients underwent surgical or medical treatment due to appropriate clinical indications. However, three patients, after being fully informed of their condition and associated risks, insisted on expectant management due to strong personal preferences. These 3 patients were closely monitored with serial assessments according to clinical guidelines [1, 3, 4], and their general condition remained stable throughout the observation period. In patient 1, fetal cardiac activity was detected in the adnexal mass during the follow-up ultrasound the next day (β-hCG from 12,558 IU/L to 16,670.4 IU/L). In patient 2, fetal cardiac activity was observed on day 4 of follow-up (β-hCG from 2477.4 IU/L to 8373.4 IU/L). In patient 3, while fetal cardiac activity was not observed, a newly formed embryonic pole was identified on ultrasound on day 2 (β-hCG from 2103 IU/L to 3496.6 IU/L). They subsequently underwent appropriate treatment.

### Correlations between serum markers and sonographic indices

The correlations between serum markers and sonographic indices in TEP are summarized in Table 2. In the total cohort, β-hCG levels were not significantly correlated with TEP mass size (*r* = 0.022, *p* = 0.69) but were positively correlated with endometrial thickness (*r* = 0.361, *p* < 0.01) and negatively correlated with the depth of pelvic free fluid (*r* = −0.208, *p* < 0.01). In simple GS-like masses, β-hCG levels were strongly positively correlated with TEP mass size (*r* = 0.720, *p* < 0.01), weakly positively correlated with endometrial thickness (*r* = 0.263, *p* < 0.01), and not significantly correlated with the depth of pelvic free fluid (*r* = −0.006, *p* = 0.94). In complicated masses, β-hCG levels were weakly positively correlated with TEP mass size (*r* = 0.211, *p* < 0.01) and endometrial thickness (*r* = 0.288, *p* < 0.01), but not correlated with the depth of pelvic free fluid (*r* = −0.022, *p* = 0.76).

**Table 2:** Correlations between serum markers and sonographic indices in TEP masses

Parameter	Total cohort (*n* = 320)		Simple GS-like mass (*n* = 128)		Complicated mass (*n* = 192)	
	*r*	*p*	*r*	*p*	*r*	*p*
β-hCG (in IU/L)						
TEP mass size (in cm)	0.022	0.69	0.720	< 0.01	0.211	< 0.01
Endometrial thickness (in cm)	0.361	< 0.01	0.263	< 0.01	0.288	< 0.01
Depth of pelvic free fluid (in cm)	−0.208	< 0.01	−0.006	0.94	−0.022	0.76
Hemoglobin (in g/L)						
TEP mass size (in cm)	−0.236	< 0.01	−0.231	0.01	−0.245	< 0.01
Endometrial thickness (in cm)	−0.013	0.82	0.083	0.35	−0.102	0.16
Depth of pelvic free fluid (in cm)	−0.246	< 0.01	−0.075	0.40	−0.344	< 0.01

*TEP* tubal ectopic pregnancy, *GS* gestational sac, *β-hCG* β-human chorionic gonadotropin

In terms of hemoglobin, a negative correlation with TEP mass size was observed in the total cohort (*r* = −0.236, *p* < 0.01), simple GS-like mass group (*r* = −0.231, *p* < 0.01), and complicated mass group (*r* = −0.245, *p* < 0.01). Hemoglobin levels were negatively correlated with the depth of pelvic free fluid in the total cohort (*r* = −0.246, *p* < 0.01) and in the complicated mass group (*r* = −0.344, *p* < 0.01), although no significant correlation was found in the simple GS-like mass group (*r* = −0.075, *p* = 0.40). No significant correlation between hemoglobin levels and endometrial thickness was found in any situation (*p* > 0.05).

**Fig. 5:**
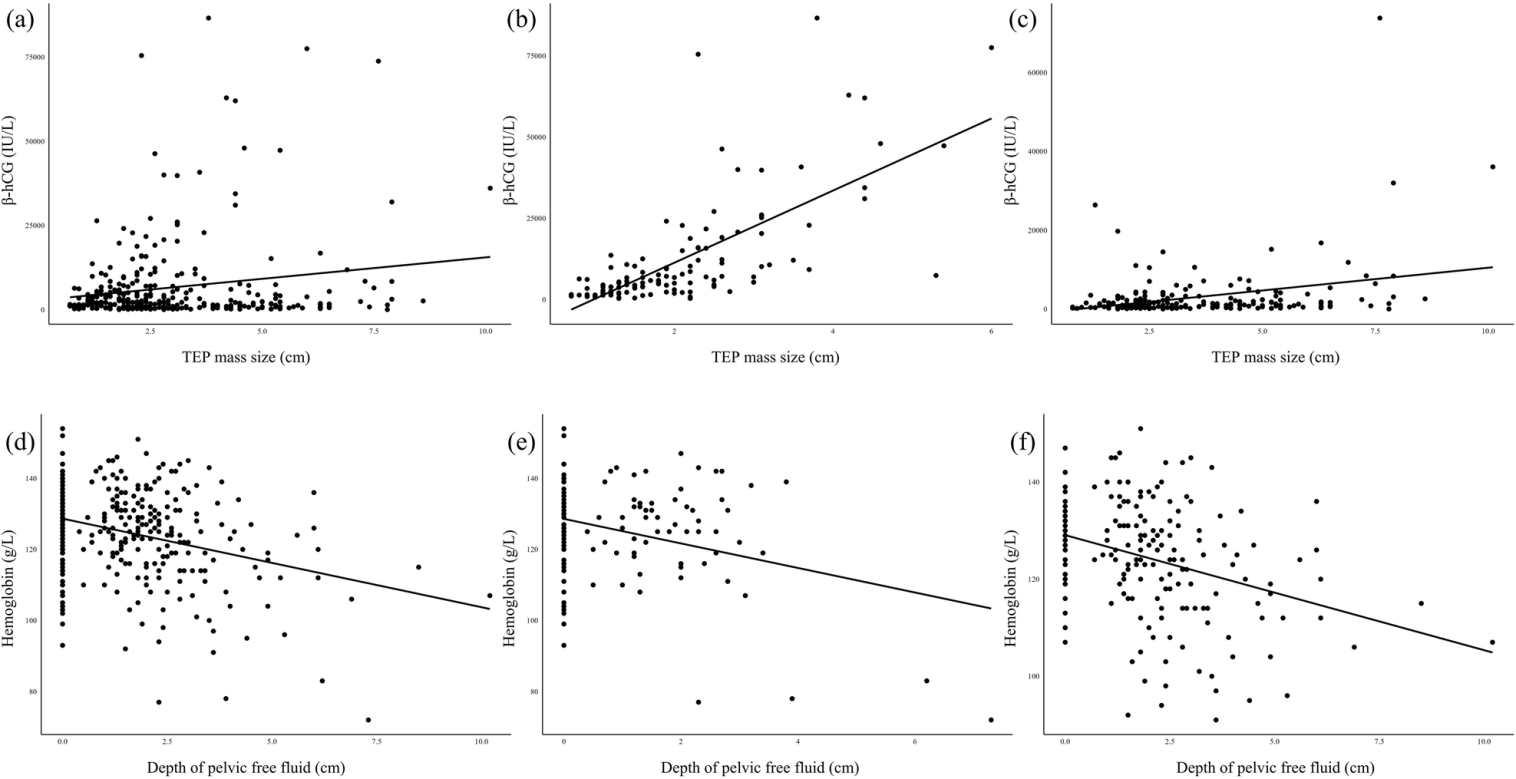
Correlations between β-hCG and TEP mass size, and between hemoglobin and the depth of pelvic free fluid. Correlations between β-hCG and mass size were analyzed in the total cohort (**a**), simple GS-like masses (**b**), and complicated masses (**c**). Correlations between hemoglobin and the depth of pelvic free fluid were also analyzed in the total cohort (**d**), simple GS-like masses (**e**), and complicated masses (**f**). β-hCG, β-human chorionic gonadotropin; TEP, tubal ectopic pregnancy; GS, gestational sac

The correlations between β-hCG and TEP mass size, and between hemoglobin and the depth of pelvic free fluid are illustrated in Fig. 5.

## Discussion

This study presents a new ultrasound-based classification for TEP. In this classification, TEPs were divided into those with simple GS-like masses and those with complicated masses, revealing the different associations between serum markers and ultrasound features in each group, which can help elucidate the pathophysiology of TEP and potentially improve management strategies.

### Pathophysiological implications

Ultrasound plays an important role in the evaluation of TEP because of its convenience, simple operation, and high sensitivity [14]. Frates et al attempted to classify TEP masses into subtypes on the basis of the presence of a yolk sac, embryo, and fetal heartbeat on ultrasound images, but the classification process lacked predictive value for clinical outcomes [12]. Similarly, Dooley et al categorized EPs into five types on the basis of variations in trophoblastic ring morphology, however, they did not consider hematosalpinx for TEP [15]. The visibility of a trophoblastic ring serves as a critical ultrasound feature in evaluating TEPs [3], however, solely focusing on this feature is often insufficient. In many cases, placental separation induces hemorrhage and consequently causes hematosalpinx and a large mass [16]. Rupture of the Fallopian tube or discharge out the fimbrial end of an intact salpinx can lead to clot formation, enveloping the Fallopian tube and rendering it unobservable on ultrasound [14, 17]. Therefore, hematosalpinx is also essential for assessing TEP.

By incorporating the clinical assessment value of these features mentioned above, we integrated them into the classification. Simple GS-like masses are characterized by clear trophoblastic ring structures, with smaller sizes, higher β-hCG levels, and higher CDFI grades, which indicate stronger pregnancy activity, as evidenced by follow-up findings in expectantly managed cases. In contrast, complicated masses accompany hematosalpinx, with lower β-hCG levels and deeper pelvic free fluid, which reflects pathological processes such as tubal abortion [18].

The correlation between β-hCG levels and mass size also reflected the difference in pregnancy activity between groups, with significant differences. In simple GS-like masses, there was a strong positive correlation, whereas in complicated masses, the correlation was weak. Trophoblastic activity is higher in the simple GS-like cases; therefore, the proliferation of trophoblastic cells directly drives mass growth, similar to the development of live normally sited pregnancies [8, 19]. In contrast, hematosalpinx in complicated ones may interfere with the linear relationship between mass size and β-hCG.

### Clinical management implications

This classification not only enhances the standardization of ultrasound evaluation but also provides more refined decision-making criteria. Although patients’ baseline demographics and symptoms were largely comparable between the two groups, the ultrasound-based categorization delineated distinct profiles that support individualized management strategies. For example, in simple GS-like TEP masses with β-hCG ≥ 5000 IU/L or masses ≥ 3.5 cm, surgery should be prioritized according to guidelines [1, 3, 4]. However, in complicated masses, mass size and β-hCG are not strongly correlated. Therefore, the importance of mass size should be weakened in complicated masses, as it may be influenced by hematosalpinx and may not actually reflect the size of the pregnancy tissue. In this situation, a more comprehensive risk assessment should be made, considering other ultrasound findings, serum markers, and clinical manifestations. Future revisions of the guidelines should incorporate type-specific indicators on the basis of the new ultrasound classification, by exploring and adjusting relevant thresholds for management across different TEP mass types.

Additionally, the positive correlation between β-hCG and endometrial thickness suggests that β-hCG may influence the endometrial response through hormonal regulation [20], but this relationship is weak. In agreement with a previous study [21], this finding suggests that endometrial thickness should not be used alone for evaluation.

In patients with complicated masses, hemoglobin levels were negatively correlated with the depth of pelvic free fluid (*r* = −0.344, *p* < 0.001), whereas no such correlation was detected in patients with simple GS-like masses. This result suggests that intra-abdominal bleeding (manifested as pelvic free fluid) in the complicated mass group can lead to systemic blood loss (lowered hemoglobin), with both indicators reflecting the severity of the condition.

Furthermore, hemoglobin levels demonstrated a consistent negative correlation with TEP mass size in both groups, with a more pronounced association observed in the complicated mass group. This suggests that larger masses in this group are likely associated with more blood loss, which may be attributed to hemorrhagic complications that arise from tubal abortion or rupture, as previously discussed in this study. Therefore, combining hemoglobin levels with the depth of pelvic free fluid can be used to more accurately assess the risk of blood loss and guide timely interventions.

### Strengths, limitations, and future directions

In this cohort study of 320 women with TEP, a novel ultrasound classification revealed two subtypes with distinct imaging and biomarker signatures as follows: simple GS-like masses presented a strong positive relationship between β-hCG levels and mass size and validated existing thresholds for surgical management, whereas complicated masses broke this link, which was likely due to hematosalpinx, making mass size alone an unreliable indicator of trophoblastic size. By combining sonographic characteristics and the serum β-hCG levels, this integrative framework allows clinicians to stratify patients more accurately. This work may serve as a valuable foundation for future prospective studies aimed at validating the proposed classification with the potential for more accurate prognostic evaluation, more refined clinical risk stratification, and a more personalized treatment approach.

Nonetheless, our findings originate from a single tertiary referral center and depend on expert radiologists, and both aspects may limit generalizability in community or resource-limited settings. Therefore, to confirm external validity and enhance clinical applicability, future work should involve multicenter, prospective validation with radiologists of varying experience, and explore the underlying biological mechanisms driving these ultrasound subtypes, with the ultimate goal of improving clinical practice.

## Conclusions

The ultrasound classification system proposed in this study integrates morphological and pathophysiological features and reveals different associations among sonographic indices and serum markers in different types of TEP. These findings not only optimize the evaluation framework for TEP but also provide key evidence for personalized treatment. In particular, although initial clinical presentations (such as abdominal pain) were comparable between the subtypes, the morphology-based classification revealed distinct profiles and management strategies. Notably, this ultrasound stratification complements existing criteria [1, 3, 4] and highlights situations where guideline-based mass size or β-hCG cutoffs should be applied with caution. Therefore, we recommend integrating this classification into future clinical guidelines to tailor management strategies (e.g., adjusting relevant thresholds for management on the basis of subtype). Future work should further validate the clinical efficacy of this classification and promote the evolution of guidelines toward precision medicine.

## Supplementary information

ELECTRONIC SUPPLEMENTARY MATERIAL

Video-S1R2.

Video-S2R2.

Video-S3R2.

Video-S4R2.

## Data Availability

The data sets analyzed during the current study are available from the corresponding author on reasonable request.

## Abbreviations

CDFI: Color Doppler flow imaging
EP: Ectopic pregnancy
GS: Gestational sac
PUMCH: Peking Union Medical College Hospital
TEP: Tubal ectopic pregnancy
β-hCG: β-Human chorionic gonadotropin
