# Supplementary material for: Novel ultrasound classification of tubal ectopic pregnancy: exploring underlying connections among sonographic and serum markers
Source: Insights Imaging. 2025 Sep 17;16:195. doi: 10.1186/s13244-025-02079-2 (PMC12443668; doi:10.1186/s13244-025-02079-2)

Novel ultrasound classification of tubal ectopic pregnancy: exploring underlying connections among sonographic and serum markers

ELECTRONIC SUPPLEMENTARY MATERIAL

Figure S1. Static frame of Video S1. This image shows a simple GS-like TEP mass. The trophoblastic ring (arrowhead) is clear and no hematosalpinx is present.

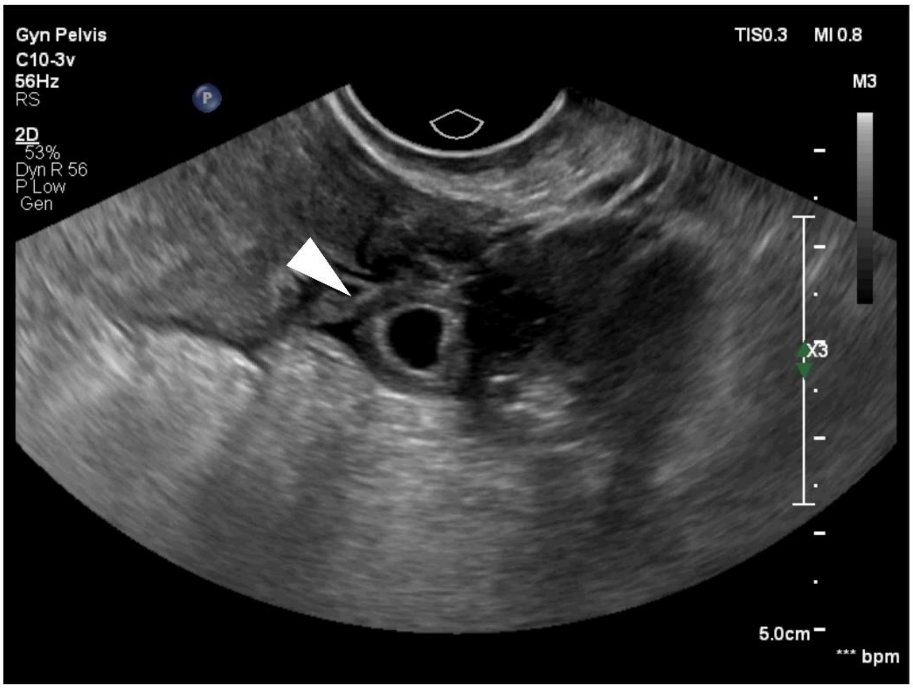

Figure S2. Static frame of Video S2. This image shows a complicated mass with both hematosalpinx (arrow) and a clear trophoblastic ring (arrowhead).

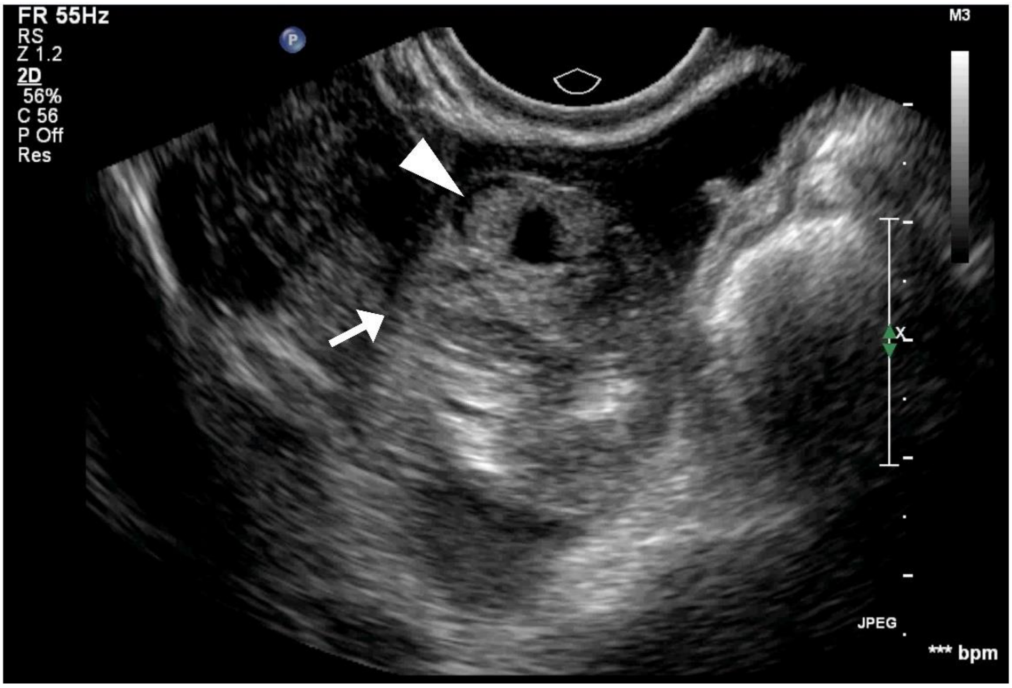

Figure S3. Static frame of Video S3. This image illustrates hematosalpinx without an obvious trophoblastic ring. The distended Fallopian tube (arrow) lacks a recognizable trophoblastic ring.

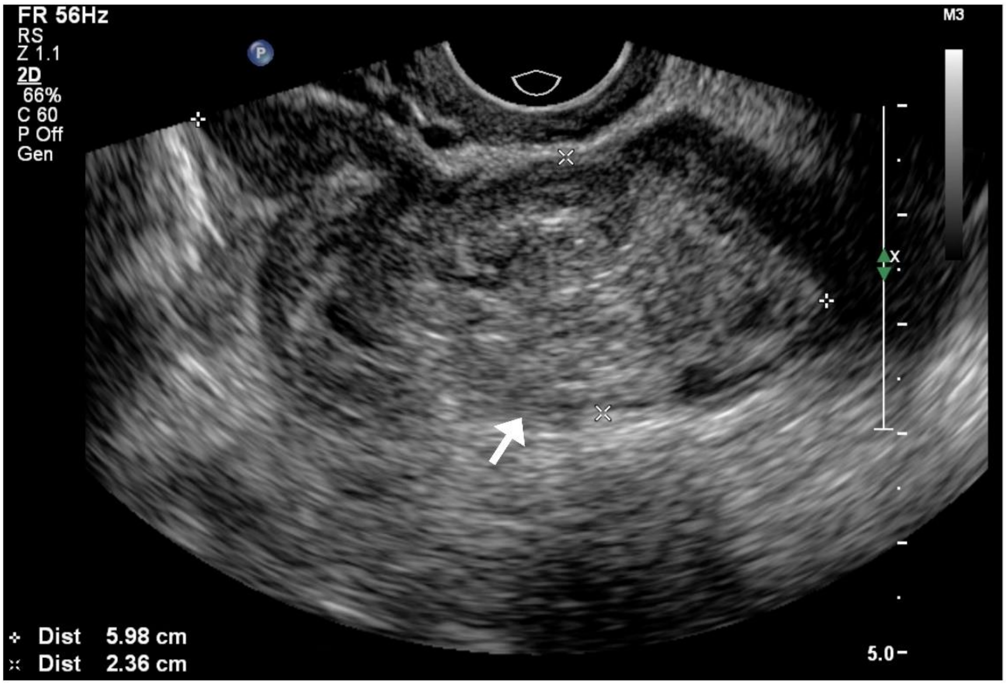

Figure S4. Static frame of Video S4. This subtype is characterized by an irregular mixed-echoic mass (arrow) and loss of recognizable tubal structure.

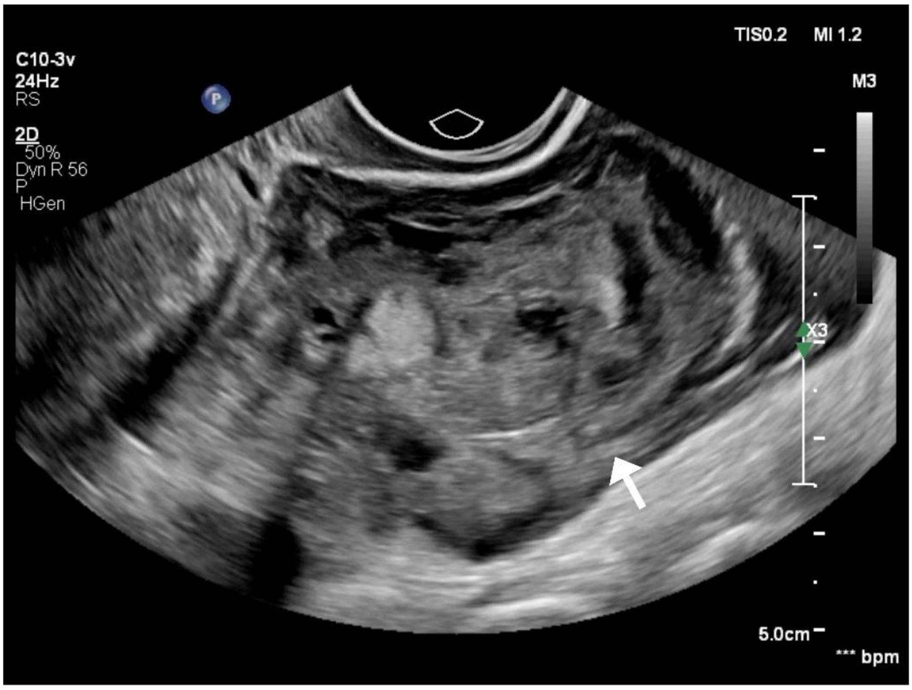

Figure S5 Color Doppler ultrasound of a complicated mass. Annular blood flow signals (arrowheads) are visible around the mass and represent blood flow of salpinx wall, whereas no intralesional flow is detected within the gestational tissue inside the mass.

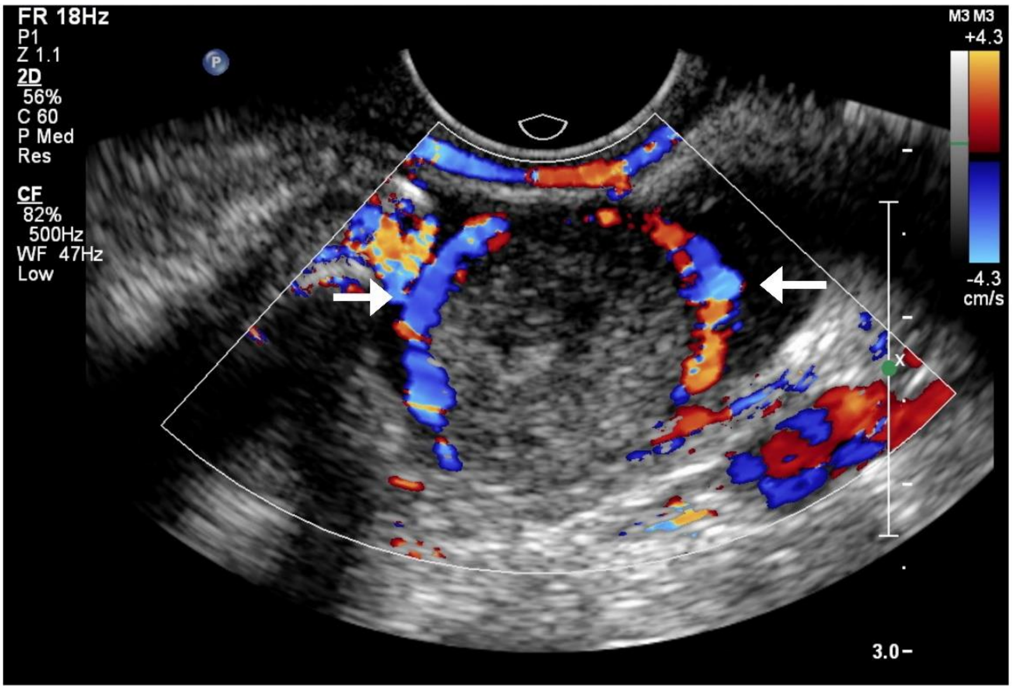

Supplement: Supplementary file 1 — ELECTRONIC SUPPLEMENTARY MATERIAL [file 13244_2025_2079_MOESM1_ESM.pdf]
